# Improving Photosynthetic Efficiency and Biochemical Adaptations in Tomatoes under Drought Stress through Seaweed Aqueous Extract Application

**DOI:** 10.1021/acsomega.5c10385

**Published:** 2026-02-25

**Authors:** Deise Munaro, Aline Nunes, Laryssa Kraufenberg, Luciana Touguinha, Gabriel Fernandes Pauletti, Joséli Schwambach, Marcelo Maraschin, Sidnei Moura

**Affiliations:** 1 LBIOP − Laboratory of Biotechnology of Natural and Synthetics Products, Technology Department, Institute of Biotechnology, 58802 University of Caxias do Sul, Caxias do Sul 95070-560, Brazil; 2 UNESP, São Paulo State University, Botucatu, São Paulo 18610-034, Brazil; 3 LBV − Laboratory of Plant Biotechnology, Institute of Biotechnology, 58802 University of Caxias do Sul, Caxias do Sul 95070-560, Brazil; 4 LESPA - Laboratory of Plant Environmental Studies, Institute of Biotechnology, University of Caxias do Sul, Caxias do Sul 95070-560, Brazil; 5 LMBA − Laboratory of Morphogenesis and Plant Biochemistry, Federal University of Santa Catarina, Florianópolis 88040-900, Brazil

## Abstract

Pursuing natural products to enhance the growth and quality of fruit and vegetable crops is crucial to ensuring sustainable food production. In this sense, this study aimed to investigate the effects of *Kappaphycus alvarezii* seaweed extract (SEA) on the physiological and biochemical responses of *Solanum lycopersicum* cv. Micro-Tom under three levels of water availability: field capacity (100% FC), moderate stress (50% MS), and severe stress (30% SS). Plants were cultivated under controlled conditions (24 ± 1 °C). Ten plants were used per treatment (*n* = 10), totaling 60 plants arranged in a completely randomized design (CRD). The treatments included a control group (C) and groups receiving 5% seaweed extract (SEA) application. The SEA was applied weekly, starting before induction of the water deficit and continuing throughout the stress period. The effects on the stomatal density, stomatal conductance, quantum yield, and morphological and biochemical parameters were evaluated. SEA-treated plants showed marked physiological and biochemical improvements under both mild and severe stress. Quantum yield increased by 7–18% compared to controls, indicating enhanced PSII photochemical efficiency. In parallel, significant increases were observed in total chlorophyll (up to 23%), soluble sugars (31%), and total flavonoids (60%) relative to those in untreated plants. These results confirm that bioactive compounds derived from seaweed can mitigate damage to the photosynthetic machinery, osmotic adjustment, and antioxidant metabolism, thereby promoting improved plant performance under limited water availability.

## Graphical abstract

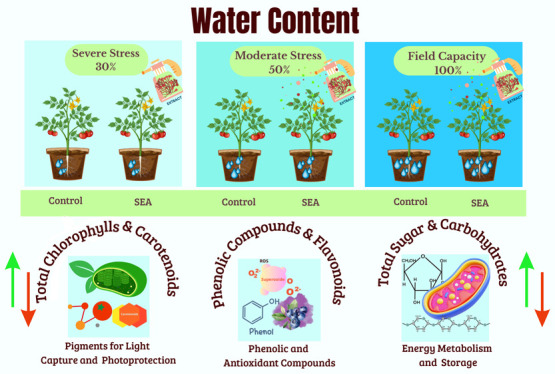

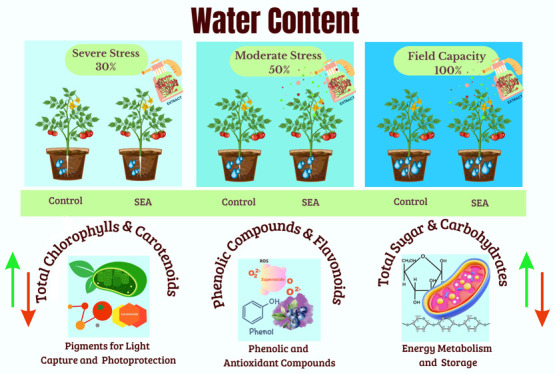

## 1 Introduction

Biostimulants are natural and/or microorganisms that, when applied to plants or soils, enhance nutrient uptake efficiency, abiotic stress tolerance, and crop quality, regardless of their nutrient content.^1^ These products encompass distinct classes such as seaweed extracts, protein hydrolysates and amino acids, humic substances, and microbial biostimulants, each characterized by specific modes of action related to metabolic modulation, signaling, and rhizosphere interactions.^1^ They act through the modulation of plant metabolism, hormone-like activity, and activation of signaling pathways related to stress defense. Seaweed extracts are among the most widely studied biostimulants due to their complex composition rich in bioactive compounds with elicitor activity.^1^

Drought stress poses an escalating threat to agricultural productivity, negatively impacting plant growth and, consequently, food production. As climate change intensifies, prolonged droughts have become increasingly common, presenting significant challenges to global food security and the economies of many countries. Climate change represents a significant threat to agricultural productivity, encompassing gradual shifts in temperature and precipitation patterns as well as a rising incidence of extreme weather events. Together, these factors exert immediate pressure on crop performance and challenge the sustainability of the current agricultural systems. This phenomenon is primarily driven by reduced rainfall, leading to extended periods of water deficiency.^2^ This leads to hydration deficits, disruptions in nutrient availability, ion accumulation to toxic levels, and increased oxidative damage.^3^

Common plant responses include reduced growth due to the downregulation of genes encoding proteins involved in cell wall expansion, DNA, and protein synthesis,^4^ as well as the inhibition of photosynthesis through the downregulation of genes from photosystems I and II, stomatal closure, damage to the photosynthetic apparatus, and chlorophyll degradation.^5,6^ As a multifaceted stress, drought affects different stages of plant development, impairing morphological, physiological, biochemical, and molecular activities.^7,8^ Among the adaptive mechanisms activated by plants are osmolyte accumulation, increased antioxidant enzyme levels,^9,10^ variations in phytohormone concentrations,^11,12^ and induction of stress response genes.^13^

In this context, identifying alternatives to support plant needs during periods of drought is crucial to the agricultural sector. One promising approach is the use of biostimulants to enhance plant tolerance under water stress. Among these strategies, the application of seaweed extracts has emerged as a viable method to mitigate the adverse effects of drought.^14^ Studies evaluating seaweed-derived extracts have reported that these extracts stimulate antioxidant enzyme activity and biosynthesis of defense metabolites, thereby reducing oxidative stress and preserving cellular function during water deficit conditions.^15–19^

However, despite the growing evidence supporting the biostimulant potential of algae extracts,^17,19,20^ most studies remain focused on agronomic outcomes, such as growth and yield, with limited mechanistic insight into the integrated physiological and biochemical responses underlying drought tolerance, particularly under controlled stress conditions and in model plant systems.

Among the macroalgae recognized as sources of potential biostimulant compounds, the red alga *Kappaphycus alvarezii* (Rhodophyta) stands out as the fifth most cultivated species worldwide. It has been shown that this species contains various compounds of interest, such as polysaccharides, amino acids, proteins, minerals, lipids, carotenoids, hormones, phenolics, and flavonoids with plant growth modulating properties. Additionally, it is the primary species used for the extraction of κ-carrageenan, an important polysaccharide widely utilized in the food and pharmaceutical industries,^20^ which has been associated with a biostimulant effect of plant growth. In a recent systematic review, Nunes et al.^21^ have reported 34 studies of this algal species published between 2017 and 2023 where improved growth and yield of crop species such as rice, maize, tomato, potato, and sugar cane have been shown. These results included not only morphophysiological increases, such as enhanced productivity and quality, but also increased tolerance to biotic and abiotic stress factors, serving as an elicitor of resistance.

Tomato was employed in this study as a model plant to explore physiological and biochemical responses to drought stress, thereby enabling mechanistic insights into biostimulant action rather than crop-specific agronomic evaluation. We hypothesized that the application of *Kappaphycus alvarezii* seaweed extract enhances drought tolerance in tomato plants by modulating key physiological and biochemical processes related to oxidative stress mitigation and water-use efficiency. The present study therefore evaluates the effectiveness of the *K. alvarezii* extract in mitigating drought-induced damage in tomato plants (*Solanum lycopersicum* cv. MicroTom), providing new insights into its biostimulant mode of action. Morphological, physiological, and biochemical traits associated with plant performance under water deficit were assessed. Overall, this study provides original mechanistic insights into the mode of action of *K. alvarezii* derived biostimulants through an integrated morphophysiological and biochemical approach under drought conditions.

## 2 Materials and Methods

### 2.1 Sample Collection

Samples of the red strain of the seaweed *Kappaphycus alvarezii* were obtained from a marine farming site located in the southern bay of Santa Catarina Island, Florianópolis, Brazil (Ribeirão da Ilha beach; 27°42′32.724″ S, 48°33′35.5″ W). Following collection, the biomass was transported under refrigerated conditions to the laboratory, rinsed with seawater to remove residual particulate matter, and subsequently processed for the preparation of the seaweed aqueous extract (SEA). The cleaned biomass was homogenized to obtain a slurry, which was then mechanically pressed and filtered to recover the soluble aqueous phase. From 1 kg of fresh biomass, approximately 83.4% of the mass was recovered as an extract solution (aqueous phase), whereas 16.6% corresponded to the residual biomass retained after filtration. The resulting extract was transferred to sterile plastic containers and stored at −20 °C until analysis and subsequent experimental use.

The chemical composition of the aqueous extract was considered equivalent to that reported by Nunes et al.,^22^ as the biomass used in the present study was collected concurrently with the material characterized by these authors. All samples originating from the same marine farming site were obtained during the same collection campaign and were subjected to identical handling and preprocessing conditions, ensuring compositional equivalence between the extracts.

The experiment was carried out in a growth chamber at 24 °C ± 1 °C, with 800 to 1200 μmol photons m^–2^·s^–1^ light intensity. Seeds of *Solanum lycopersicum* cv. MicroTom were sown in 200 mL pots filled with a Carolina Soil commercial substrate. Ten plants were analyzed per treatment in a completely randomized design, involving 60 plants.

SEA was applied weekly during the vegetative phenological stage along with a Hoagland nutrient solution recommended for the crop. At the flowering stage and during the maturation of the first truss, plants were subjected to water deficit following the method adapted from Ruiz-Lozano et al.^20^ To induce water stress, the substrate mass in the pots was standardized and the water retention capacity of the substrate relative to the container was calculated using the retention curve, enabling the establishment of three water availability conditions. The experiment included six treatments, combining water stress levels and the application of SEA or a control treatment (C).

The water stress levels were defined based on the water retention capacity of the substrate as follows: (i) field capacity (100%, FC), (ii) moderate stress (50% MS), and (iii) severe stress (30% SS). The water stress treatments were initiated when 50% of the plants reached the flowering phenological stage. Water replacement was performed daily until the soil reached the moisture at FC for the nonstressed condition, while for the water deficit condition, the water stress was induced by daily replenishment of water to achieve the desired FC, which was replenished every 2 days based on the calculated water deficit. For each stress level, two groups were established: one receiving no SEA extract (control, C) and the other treated with a 5% SEA solution.

Thus, the six treatments were as follows: FC-C: field capacity with no seaweed extract; FC-SEA: field capacity with seaweed extract; MS-C: moderate water stress without seaweed extract; MS-SEA: moderate water stress with seaweed extract; SS-C: severe water stress with no seaweed extract; SS-SEA: severe water stress with seaweed extract.

Foliar application of the 5% SEA solution or water (control) was performed weekly, ensuring full coverage of the leaf surface until runoff. Applications began before the induction of water stress and continued throughout the experiment.

### 2.2 Morphophysiological Analysis

Plant growth and metabolic responses were evaluated throughout the stress period, with continuous measurements of stomatal conductance and quantum yield (*n* = 5) with three measurements at the medial region of plants. At the end of the experiment, growth parameters were measured, including plant height, number of flowers, and number of fruits in the first, second, and third trusses.

#### 2.2.1 Stomatal Density

Slides for stomatal counting were prepared by applying a clear nail polish to the adaxial leaf surface. After drying, adhesive tape was used to remove a “negative” of the epidermis, which was then transferred to a glass slide with or without glycerin. Stomatal density (*D*) was calculated using the formula *D* = number of stomata/0.272902 mm^2^, based on images captured with 100× objective lens and recorded in a spreadsheet, using a light optical microscope (Leica, DM250, Leica Microsystems, Wetzlar, Germany) described elsewhere.^23^ For each repetition, one slide with approximately five sections was prepared and three random images were captured per slide. Images of leaves were captured against a calibrated scale and analyzed using ImageJ software to calculate the number of stomata.

#### 2.2.2 Stomatal Conductance

Stomatal conductance was measured using a method adapted from Murray et al.^24^ with a steady-state leaf porometer (Leaf Porometer upgraded model SC-1, 2011, Decagon Devices). For each treatment, five plants were selected, and two leaves were measured per plant. Fully grown green leaves on the same side of all of the sampled plants were measured twice each week during the stress period.

#### 2.2.3 Quantum Yield Measurements

The maximum quantum yield of the PSII photosystem (Fv/Fm = (Fm – F0)/Fm) was determined using a fluorometer (FluorPen FP 100, Drasov, Czech Republic), following the method adapted from Todorova et al.^25^ The initial fluorescence (F0) was measured in dark-adapted samples, followed by the maximum fluorescence (Fm) after exposure to a saturating light pulse (λ = 625 nm, 12 μmol of photons m^–2^·s^–1^). The pulse intensity was adjusted to 50–70%, as recommended by the manufacturer, to ensure PSII saturation without inducing plant stress. Dark adaptation was performed for at least 30 min before measurement to allow stabilization of PSII reaction centers. Fv/Fm values were expressed as a percentage, following the meter’s scale, which ranges from 0 to 1. Three measurements were taken per plant, with a total of five plants per treatment. Measurements were conducted twice a week during the water stress period, resulting in a total of four readings per plant.

#### 2.2.4 Productivity Analysis

To measure plant height, 10 plants from each treatment were assessed by using a graduated ruler, measuring from the base to the apex. Additionally, the number of flowers and fruits was counted manually at the end of the development period, approximately 70 days after sowing.

### 2.3 Biochemical Analysis

For the biochemical analyses, two leaves were collected from the middle third, weighed on an analytical balance, and macerated using liquid nitrogen. The contents of chlorophyll *a* (Chl a), chlorophyll *b* (Chl b), total chlorophyll (Chl total), total carotenoids (TCN), total soluble sugars (TSS), total starch (TS), total carbohydrates (TC), total phenolic content (TPC), and total flavonoid content (TFC) were determined. The experiment included three true replications per treatment with each analysis performed in triplicate, totaling nine repetitions per treatment. On the day of collection, the leaves were weighed for all analyses and stored in Falcon tubes at −20 °C until further analysis.

#### 2.3.1 Chlorophyll (Chl) and Total Carotenoids (TCN)

Leaf samples weighing 100 mg were incubated in a water bath with 7 mL of dimethyl sulfoxide (DMSO, Sigma-Aldrich, USA) for 2 h at 65 °C. The extract was then collected through filtration, and the final volume was adjusted to 10 mL with DMSO, following the protocol established by Hiscox and Israelstam.^26^ Absorbance measurements were taken at 480, 649, and 665 nm by using a UV–vis spectrophotometer (Bel Spectro LGS53, Bel Engineering, Monza, Italy). The equations described by Hiscox and Israelstam^26^ were applied to Chl absorbance values, and the results were expressed in mg/g:

Chla=[12.19x(A665)−3.45x(A649)]

Chlb=[21.99x(A649)−5.32x(A665)]

To quantify TCN in the DMSO extract, the following formula was utilized:

$$\mathrm{TCN}=\frac{\left[1000x(A480)-2.14x(\mathrm{Chl}\;a)-70.16x(\mathrm{Chl}\;b)\right]}{220}$$

#### 2.3.2 Total Soluble Sugars (TSS)

The determination of TSS used a method modified from Umbreit et al.^27^ In summary, 50 mg of leaves was extracted with a 2 mL solution of methanol, chloroform, and water (MCW – 12:5:3, v/v/v). The mixture was centrifuged (4000 rpm, 10 min), and the supernatant was collected. A second extraction was performed on the remaining pellet, and the supernatants were pooled. The upper aqueous phase was isolated, and 2 mL of a 0.2% anthrone (Sigma-Aldrich, USA) solution in sulfuric acid was added and vortexed. This solution was heated in a water bath at 100 °C for 3 min and allowed to cool to room temperature. Absorbance was measured at 630 nm by using a UV–vis spectrophotometer (SpectraMax 190 Microplate Reader, Molecular Devices, California, USA). A standard curve for glucose Sigma-Aldrich, MO, USA) was built with concentrations ranging from 62.50 to 2000 μg/mL (*y* = 0.0018*x*, *r* ^2^ = 0.9517). Absorbance values and the results were expressed in μg/mL.

#### 2.3.3 Total Starch (TS)

The TS content was determined using a method adapted from Umbreit et al.^27^ The remaining pellet from the TSS analysis (Section 2.3.2) was extracted with 2 mL of 30% perchloric acid (Sigma-Aldrich, USA), followed by centrifugation (4000 rpm, 10 min) and the collection of the supernatant. This extraction was performed a second time, and the supernatants were pooled. An aliquot (1 mL of supernatant) was mixed with 2 mL of a 0.2% anthrone solution in sulfuric acid and vortexed. The mixture was then heated (water bath, 100 °C, 3 min) and allowed to cool to room temperature. Absorbance was measured at 630 nm using a microplate reader (ThermoPlate, model P-reader). A calibration curve was established using starch (Sigma-Aldrich, MO, USA) as the analytical standard at concentrations ranging from 62.50 to 2000 μg/mL (*y* = 0.0016*x*, *r* ^2^ = 0.9916).

#### 2.3.4 Total Carbohydrates (TC)

The TC content was determined using a method adapted from Du Bois et al.,^28^ with modifications as follows: 200 mg of leaf tissue was combined with 20 mL of distilled water and then centrifuged (4000 rpm, 10 min). From the resulting supernatant, 2 mL was collected and added with 0.05 mL of 80% phenol and 5 mL of concentrated sulfuric acid. After a 10 min incubation, the mixture was vortexed and then transferred to a water bath at 30 °C for 15 min. Absorbance was read at 490 nm using a UV–vis spectrophotometer (SpectraMax 190 Microplate Reader, Molecular Devices, California, USA). For TC calculation, a calibration curve was built using galactose (Sigma-Aldrich, MO, USA) as analytical standard at concentrations ranging from 7.80 to 500 μg/mL (*y* = 0.0025*x*, *r* ^2^ = 0.9992). Absorbance values and the results were expressed in μg/mL.

#### 2.3.5 Total Phenolic Content (TPC)

The methodology of Singleton et al.^29^ was used for the TPC determination, with adaptations. Briefly, 300 mg of leaf sample was weighed and mixed with 6 mL of 80% methanol. The mixture was kept in the dark for 1 h. Afterward, the samples were centrifuged at room temperature for 5 min at 4000 rpm, and 100 μL of the supernatant was collected. In a Falcon tube, 75 μL of the Folin–Ciocalteu reagent (Sigma-Aldrich, MO, USA) and 825 μL of 2% (w/v) sodium carbonate solution were added to the supernatant. This mixture was incubated in the dark for 1 h. Subsequently, 300 μL of the sample was transferred to microplates (*n* = 3) and the absorbance was measured at 750 nm using a microplate reader (ThermoPlate, model P-reader). Gallic acid (Sigma-Aldrich, MO, USA) was utilized as the analytical standard (7.81 to 500 μg/mL) for building a standard curve (*y* = 0.006*x*, *r* ^2^ = 0.9967) for the determination of TPC. Absorbance values and the results were expressed in μg/mL.

#### 2.3.6 Total Flavonoid Content (TFC)

The TFC was determined using a method adapted from Woisky and Salatino.^30^ From the supernatant obtained during the total phenolic content (TPC) analysis (Section 2.3.4), 500 μL was collected and mixed with 2.5 mL of absolute ethanol and 500 μL of 2% aluminum chloride solution in methanol. The mixture was vortexed and incubated in the dark for 1 h. Absorbance was measured at 420 nm using a microplate reader (ThermoPlate, model P-reader) with 300 μL of the sample (*n* = 3). A calibration curve was created using quercetin as the analytical standard (Sigma-Aldrich, MO, USA) at concentrations ranging from 7.81 to 500 μg/mL (*y* = 0.0056*x*, *r* ^2^ = 0.9799). Absorbance values and the results were expressed in μg/mL.

### 2.4 Statistical Analysis

For the morphophysiological and biochemical data sets, data normality and homogeneity of variances were previously evaluated using the Shapiro–Wilk and Levene tests, respectively. Data were then analyzed using one-way analysis of variance (ANOVA), considering treatment as the single fixed factor. When significant differences were detected, means were compared using the Scott–Knott test (*p* < 0.05), with a 5% error probability, using AgroEstat software (v. 1.1.0.712). Additionally, the morphophysiological and biochemical parameters were analyzed through principal component analysis (PCA) using the singular value decomposition (SVD) algorithm, performed with the support of Unscrambler X software (v. 10.4).

## 3 Results

### 3.1 Morphophysiological Parameters

The measurements of plant height, number of flowers, and number of fruits of the biostimulant-treated plants did not differ (*p* < 0.05) from the control under all conditions of water stress investigated (Table 1).

**Table 1:** Plant Height (cm), Number of Fruits, and Number of Flowers of Tomato Plants Cultivated under Different Water Stress Conditions with and without the Use of the *Kappaphycus alvarezii* Biostimulant^a^

treatment	plant height	number of fruits	number of flowers
FC-C	19.70 ± 3.68^ns^	0.56 ± 0.53^ns^	2.44 ± 1.42^ns^
FC-SEA	20.18 ± 3.70	1.44 ± 1.01	2.44 ± 2.74
MS-C	20.13 ± 2.38	1.67 ± 1.00	2.67 ± 2.74
MS-SEA	23.20 ± 2.67	1.56 ± 1.42	4.78 ± 2.54
SS-C	18.43 ± 4.93	1.33 ± 1.32	3.22 ± 3.67
SS-SEA	22.29 ± 5.11	1.56 ± 1.33	1.22 ± 1.99

a ns – not significant by the Scott & Knott test (*p* < 0.05). FC-C – Field capacity with no seaweed extract; FC-SEA – Field capacity with seaweed extract; MS-C – Moderate water stress with no seaweed extract; MS-SEA – Moderate water stress with seaweed extract; SS-C – Severe water stress with no seaweed extract; SS-SEA – Severe water stress with seaweed extract.

Regarding the stomatal density measurements, no significant differences were found between the control plants and those treated with the algal extract over the sampling days for both adaxial and abaxial leaf surfaces (Table 2).

**Table 2:** Stomatal Conductance (mmol m^–2^ s^–1^) Measured on Adaxial and Abaxial Leaf Surfaces of Tomato Plants Grown under Different Water Stress Conditions with and without *Kappaphycus alvarezii* Biostimulant Application^a^

	day 01		day 02		day 03		day 04	
treatment	adaxial	abaxial	adaxial	abaxial	adaxial	abaxial	adaxial	abaxial
FC-C	15.66 ± 4.53^ns^	25.44 ± 7.07^ns^	16.38 ± 7.72^ns^	54.34 ± 16.30^ns^	15.54 ± 5.95^ns^	32.60 ± 19.86^ns^	14.82 ± 2.65^ns^	39.14 ± 10.78^ns^
FC-SEA	14.60 ± 3.43	30.02 ± 12.51	16.92 ± 4.02	43.02 ± 7.87	18.72 ± 8.45	35.52 ± 8.96	21.08 ± 6.93	33.34 ± 4.27
MS-C	19.12 ± 16.24	21.46 ± 8.64	23.22 ± 12.31	52.10 ± 18.57	24.66 ± 5.95	26.62 ± 7.99	29.46 ± 22.02	52.14 ± 8.51
MS-SEA	26.32 ± 6.23	37.14 ± 11.86	16.04 ± 3.27	52.18 ± 6.15	26.16 ± 14.20	29.54 ± 8.60	26.70 ± 4.79	54.92 ± 9.08
SS-C	17.28 ± 7.22	24.08 ± 8.73	21.28 ± 5.77	45.30 ± 7.88	14.08 ± 6.68	46.22 ± 24.23	18.84 ± 8.47	47.74 ± 21.42
SS-SEA	20.32 ± 6.89	32.30 ± 7.11	26.48 ± 15.10	53.66 ± 26.01	14.40 ± 4.00	51.62 ± 17.99	36.64 ± 13.15	55.82 ± 22.04

a ns – not significant by the Scott & Knott test (*p* < 0.05). FC-C – Field capacity with no seaweed extract; FC-SEA – Field capacity with seaweed extract; MS-C – Moderate water stress with no seaweed extract; MS-SEA – Moderate water stress with seaweed extract; SS-C – Severe water stress with no seaweed extract; SS-SEA – Severe water stress with seaweed extract.

For the quantum yield results, measurements on days 1, 2, and 4 showed statistical differences between the treatments only on the third day, where the MS-SEA and SS-SEA treatments exhibited the highest values, with 0.61 and 0.65, respectively, on a scale from 0 to 1 (Table 3).

**Table 3:** Quantum Yield (Scale from 0 to 1) of Tomato Plants Cultivated under Different Water Stress Conditions with and without the Use of the *Kappaphycus alvarezii* Biostimulant^a^,^b^

treatment	day 01	day 02	day 03	day 04
FC-C	0.65 ± 0.05^ns^	0.58 ± 0.06^ns^	0.56 ± 0.06 b	0.60 ± 0.09^ns^
FC-SEA	0.61 ± 0.08	0.61 ± 0.08	0.57 ± 0.06 b	0.64 ± 0.07
MS-C	0.62 ± 0.08	0.60 ± 0.08	0.59 ± 0.07 b	0.63 ± 0.07
MS-SEA	0.65 ± 0.07	0.64 ± 0.07	0.61 ± 0.06 a	0.64 ± 0.08
SS-C	0.59 ± 0.05	0.59 ± 0.06	0.57 ± 0.06 b	0.62 ± 0.06
SS-SEA	0.65 ± 0.06	0.62 ± 0.07	0.65 ± 0.05 a	0.61 ± 0.07

a Means followed by different letters in the columns differ statistically using the Scott & Knott test (*p* < 0.05).

b ns – not significant by the Scott & Knott test (*p* < 0.05). FC-C – Field capacity with no seaweed extract; FC-SEA – Field capacity with seaweed extract; MS-C – Moderate water stress with no seaweed extract; MS-SEA – Moderate water stress with seaweed extract; SS-C – Severe water stress with no seaweed extract; SS-SEA – Severe water stress with seaweed extract.

The principal component analysis (PCA) revealed that PC1 and PC2 together explained 68% of the total variance in the data set (Figure 1). The samples formed distinct clusters according to the treatments. One cluster comprised SS-C and FC-C (PC1 + , PC2 – ), while FC-SEA appeared as a separate cluster positioned in a different quadrant (PC1 + , PC2 + ). These three treatments were far from the morphophysiological variables analyzed. In contrast, SS-SEA and MS-SEA each formed independent clusters associated with different sets of variables. The SS-SEA cluster (PC1 – and PC2 – ) was grouped with quantum yield parameters (day 1 and day 3), abaxial stomatal conductance (day 2 and day 4), and adaxial stomatal conductance (day 2 and day 4). The MS-SEA cluster (PC1 – , PC2 + ) was associated with plant height, number of fruits, number of leaves, quantum yield (days 2 and 4), abaxial stomatal conductance (day 1), and adaxial stomatal conductance (day 1 and day 3).

**Figure 1:**
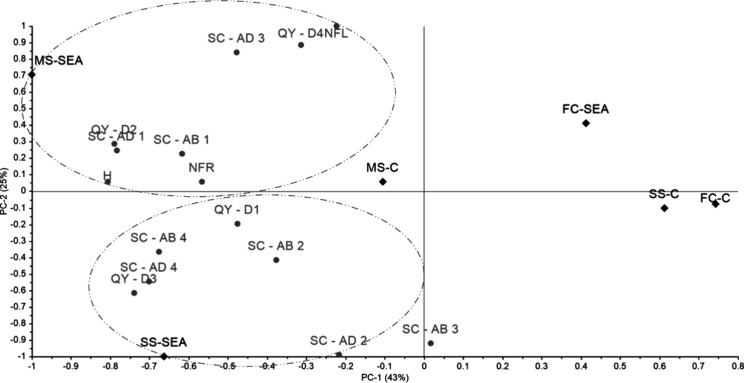
Principal component analysis (PCA) of the morphophysiological data set of tomato plants cultivated under different water stress conditions, with and without the use of the *Kappaphycus alvarezii* biostimulant. H – height; NFR – number of fruits; NFL – number of flowers; QY – quantum yield (days 1, 2, 3, and 4); SC – stomatal conductance (days 1, 2, 3, and 4); AD – adaxial; AB – abaxial; FC-C – Field capacity with no seaweed extract; FC-SEA – Field capacity with seaweed extract; MS-C – Moderate water stress with no seaweed extract; MS-SEA – Moderate water stress with seaweed extract; SS-C – Severe water stress with no seaweed extract; SS-SEA – Severe water stress with seaweed extract.

### 3.2 Biochemical Analysis

Total contents of Chl a, Chl b, and total Chl differed in plants treated with FC-SEA, MS-SEA, and SS-C with respect to the others. For the FC-SEA and MS-SEA treatments, increases by 22% and 27% in total chlorophyll were detected in comparison to control plants. Contrarily, it was found that SS-SEA-treated plants presented a total chlorophyll content 30% lower as compared to control, i.e., SS-C (Table 4). Additionally, a higher TCN content was recorded for the MS-C, MS-SEA, and SS-C treatments. Although the differences from the other treatments were minimal, they were statistically significant (Table 4).

**Table 4:** Chlorophyll a mg g^–1^, Chlorophyll b mg g^–1^, Total Chlorophyll mg g^–1^, and Total Carotenoid mg g^–1^ Concentrations of Tomato Plants Cultivated under Different Water Stress Conditions with and without the Use of the *Kappaphycus alvarezii* Biostimulant^a^

treatment	chlorophyll a	chlorophyll b	total chlorophyll	total carotenoid
FC-C	0.62 ± 0.11 b	0.29 ± 0.05 b	0.91 ± 0.16 b	0.07 ± 0.00 b
FC-SEA	0.76 ± 0.04 a	0.35 ± 0.02 a	1.11 ± 0.06 a	0.07 ± 0.01 b
MS-C	0.60 ± 0.01 b	0.28 ± 0.01 b	0.88 ± 0.02 b	0.08 ± 0.00 a
MS-SEA	0.76 ± 0.09 a	0.36 ± 0.05 a	1.12 ± 0.14 a	0.09 ± 0.00 a
SS-C	0.77 ± 0.08 a	0.36 ± 0.04 a	1.13 ± 0.13 a	0.08 ± 0.01 a
SS-SEA	0.53 ± 0.14 b	0.24 ± 0.07 b	0.78 ± 0.21 b	0.07 ± 0.01 b

a Means followed by different letters in the columns differ statistically using the Scott & Knott test (*p* < 0.05). FC-C – Field capacity with no seaweed extract; FC-SEA – Field capacity with seaweed extract; MS-C – Moderate water stress with no seaweed extract; MS-SEA – Moderate water stress with seaweed extract; SS-C – Severe water stress with no seaweed extract; SS-SEA – Severe water stress with seaweed extract.

In the TSS content, a higher level (*p* < 0.05) was observed for the SS-SEA treatment (67.43 g/g), with a 42% increase compared to SS-C (47.51 mg/g), also statistically differing from the other treatments. The analysis of TS revealed a superior amount for the SS-C-treated plants (27.82 mg/g), differing from the other treatments. For TC, the highest content was observed in MS-SEA (43.63 mg/g), representing a 30% increase compared to MS-C (33.57 mg/g). This value was significantly different from those of both MS-C and the other treatments (Table 5).

**Table 5:** Total Soluble Sugars mg g^–1^, TSS, Total Starch mg g^–1^, TS, and Total Carbohydrate mg g^–1^, TC, Contents of Tomato Plants Cultivated under Different Water Stress Conditions with and without the Use of the *Kappaphycus alvarezii* Biostimulant^a^

treatment	total soluble sugars	total starch	total carbohydrate
FC-C	51.56 ± 2.76 c	21.97 ± 1.12 b	33.47 ± 3.65 b
FC-SEA	53.73 ± 2.87 c	16.08 ± 1.17 c	27.28 ± 6.34 c
MS-C	59.01 ± 3.54 b	15.52 ± 1.21 c	33.57 ± 4.86 b
MS-SEA	62.67 ± 6.83 b	17.94 ± 1.33 c	43.63 ± 11.64 a
SS-C	47.51 ± 3.78 c	27.82 ± 5.51 a	19.31 ± 4.47 c
SS-SEA	67.43 ± 7.76 a	13.68 ± 2.39 c	36.59 ± 6.96 b

a Means followed by different letters in the columns differ statistically using the Scott & Knott test (*p* < 0.05). FC-C – Field capacity with no seaweed extract; FC-SEA – Field capacity with seaweed extract; MS-C – Moderate water stress with no seaweed extract; MS-SEA – Moderate water stress with seaweed extract; SS-C – Severe water stress with no seaweed extract; SS-SEA – Severe water stress with seaweed extract.

Regarding TPC and TFC contents, the results were similar across treatments. For TPC, the highest value was observed in the FC-C treatment (4.65 mg/g), which differed statistically from the others. For TFC, FC-C also showed the highest content (16.46 mg/g), which was not statistically different from FC-SEA (16.37 mg/g) but was significantly different from the remaining treatments (Table 6).

**Table 6:** Total Phenolic mg g^–1^ TPC and Total Flavonoid mg g^–1^ TFC Contents of Tomato Plants Cultivated under Different Water Stress Conditions with and without the Use of the *Kappaphycus alvarezii* Biostimulant^a^

treatment	TPC	TFC
FC-C	4.65 ± 1.09 a	16.46 ± 4.13 a
FC-SEA	3.78 ± 1.05 b	16.37 ± 6.60 a
MS-C	3.40 ± 0.36 b	10.77 ± 0.45 b
MS-SEA	3.63 ± 0.20 b	12.23 ± 1.35 b
SS-C	3.29 ± 0.09 b	10.77 ± 1.22 b
SS-SEA	3.04 ± 0.52 b	9.82 ± 0.56 b

a Means followed by different letters in the columns differ statistically using the Scott & Knott test (*p* < 0.05). FC-C – Field capacity with no seaweed extract; FC-SEA – Field capacity with seaweed extract; MS-C – Moderate water stress with no seaweed extract; MS-SEA – Moderate water stress with seaweed extract; SS-C – Severe water stress with no seaweed extract; SS-SEA – Severe water stress with seaweed extract.

PCA was performed on the biochemical data set of tomato plants treated with the algal biostimulant. The first two components explained 75% of the total variance, with PC1 accounting for 50% and PC2 accounting for 25% (Figure 2). Distinct groupings were observed: SS-SEA and MS-C clustered with TSS and TC (PC1 – , PC2 + ); MS-SEA clustered with TCN (PC1 + , PC2 + ); SS-C clustered with Chl a, Chl b, total chlorophyll, and TS (PC1 + , PC2 + ); and both FC-SEA and FC-C clustered with TPC and TFC (PC1 + , PC2).

**Figure 2:**
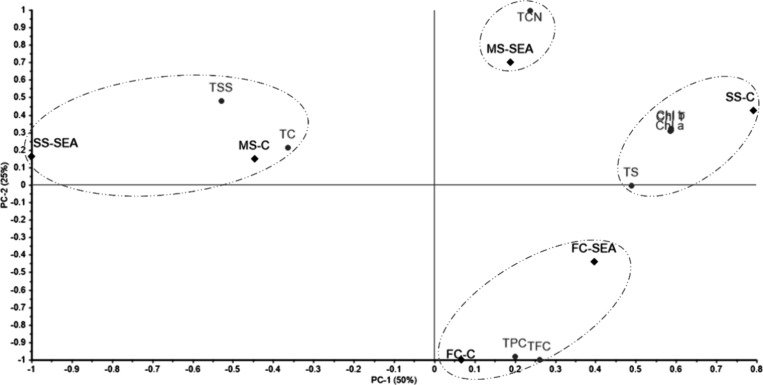
Principal component analysis (PCA) of the biochemical data set of tomato plants cultivated under different water stress conditions with and without the use of the *Kappaphycus alvarezii* biostimulant. Chl a – Chlorophyll a; Chl b – Chlorophyll b; Chl total – Total chlorophyll; TCN – Total carotenoids; TSS – Total soluble sugars; TS – Total starch; TC – Total carbohydrates; TPC – Total phenolic content; TFC – Total flavonoid content; FC-C – Field capacity with no seaweed extract; FC-SEA – Field capacity with seaweed extract; MS-C – Moderate water stress with no seaweed extract; MS-SEA – Moderate water stress with seaweed extract; SS-C – Severe water stress with no seaweed extract; SS-SEA – Severe water stress with seaweed extract.

## 4 Discussion

Foliar application of the biostimulant containing *K. alvarezii* extract did not significantly improve the morphological parameters (Table 1). Likewise, stomatal conductance showed no significant differences between the control and the extract treatments. In contrast, biochemical parameters (Table 2) differed significantly among applications under distinct water stress conditions.

Taken together, these results indicate that drought stress primarily affected plant metabolism rather than morphology and that the application of *K. alvarezii* extract modulated this metabolic plasticity in a stress-intensity-dependent manner. While morphological traits remained relatively stable within the experimental time frame, biochemical and physiological adjustments reveal coordinated responses that underpin plant acclimation to water deficit.

These responses reflected differential adjustments in primary and secondary metabolism, indicating both defense activation and basic resource maintenance by the plants. Under nonstress or milder environmental conditions, plants treated with *Kappaphycus alvarezii* extract tended to channel metabolic resources toward the enhancement of flavonoid and phenolic compound biosynthesis.

These pathways, associated with secondary metabolism, are strongly linked to antioxidant activity and play essential roles in modulating plant responses to biotic and abiotic factors. This pattern was observed under field capacity conditions. In contrast, plants subjected to moderate (MS) and severe (SS) drought stress activated metabolic routes that increased the accumulation of primary and secondary metabolites, which is crucial for maintaining fundamental physiological processes and ensuring survival under adverse environmental conditions. For example, total chlorophylls and carotenoids increased by up to 1.23 times in MS-SEA and 1.29 times in SS-SEA compared to those in the control. Primary metabolism-related parameters also showed expressive adjustments induced by the extract under a water deficit. Total soluble sugars increased from 51.56 in the control to 67.43 in the SS-SEA, representing a 31% rise. Similarly, the total amount of carbohydrates increased from 33.4 in the control to 43.16 in MS-SEA, a 29% increase. Together, these increases indicate that plants treated with the extract under drought conditions intensify pathways associated with energy maintenance, accumulation of compatible osmolytes, and cellular protection, contributing to the preservation of homeostasis and the maintenance of essential physiological functions during stress.

These metabolic adjustments are closely linked to physiological performance, as the maintenance of photosynthetic pigments and the accumulation of soluble sugars directly support energy balance, osmotic regulation, and protection of the photosynthetic apparatus under drought conditions.

Physiological parameters, such as plant height and the number of fruits and flowers, provide valuable insights into the adaptive functional state of plants in response to environmental stressors or treatments. Consequently, biochemical and physiological parameters may reveal metabolic deregulations or the activation of alternative pathways. These initial observations are essential for understanding the underlying mechanisms that influence the agronomic responses of plants, including productivity. Therefore, it is possible that the measurements did not show differences among stress conditions within the time frame designated for analysis.^31^

Furthermore, research highlights that plant resistance to stress factors is a complex trait resulting from changes at the molecular, metabolic, and physiological levels.^32^ The primary strategy plants use to cope with stress is their ability to survive, recover biochemical functions, and promote reproductive induction,^33^ a pattern that is consistent with the responses observed in this study.

Drought stress imposes simultaneous limitations on carbon assimilation and water availability, requiring coordinated anatomical, physiological, and metabolic adjustments. In this context, stomatal traits play a central role by linking water status to photosynthetic efficiency and metabolic demand.

The increase in stomatal density under severe stress may be related to a physiological adjustment mechanism aimed at optimizing gas exchange and enhancing water use efficiency in adverse conditions. Studies indicate that stomatal density and size are influenced by environmental factors, including water and nutrient availability, and are modulated by systemic signals from mature leaves that detect environmental changes.^30,31^ In this sense, the stomatal density under water deficit can vary based on the severity of stress, so that an increase may occur under moderate stress, followed by a potential reduction under extreme stress.^34–36^ This behavior may be linked to adaptive strategies for maintaining water homeostasis and optimizing CO_2_ assimilation when water and nutrient availability is limited.

Chua and Lau^37^ indicate that some plant species, including tomatoes, can respond to water stress with an increase in stomatal density, often accompanied by a reduction in stomatal size. This mechanism could facilitate faster responses to environmental fluctuations and improve the water use efficiency. Studies by Zhao et al.,^38^ Zhou et al.,^39^ Song et al.,^40^ and Driesen et al.^41^ have demonstrated this anatomical and physiological pattern across various species, including wheat, rice, basil, and tomato, reinforcing the hypothesis that such strategies contribute to adaptation to water stress.

Our findings corroborate this trend as tomato plants under severe stress (SS) conditions showed a marked increase in stomatal density, in line with previous reports. This result suggests that under extreme water stress, stomatal plasticity may act as an adaptive mechanism to maintain gas exchange and minimize impacts on photosynthesis, aligning with observations from other studies. Thus, our data provide evidence that stomatal regulation in tomatoes under drought may follow patterns similar to those described for other crop species, serving as a key factor in drought tolerance.

Regarding the higher quantum yield values, it was observed that they are associated with greater accumulation of carotenoids, particularly in the MS-SEA treatment. The treatment with algae extracts under moderate stress conditions not only helped mitigate photochemical impairment and reduce stress-induced damage to photosystem efficiency but also exhibited superior values compared to the control and to the field capacity (FC-C) treatment for total chlorophyll and carotenoid concentrations.

The positive association between a higher quantum yield and increased carotenoid content suggests that the biostimulant contributes to photoprotection by enhancing antioxidant capacity, thereby preserving PSII efficiency under moderate drought stress.

The most common parameters used to assess abiotic interference in the photosynthetic process include the maximum photochemical quantum yield of PSII (Fv/Fm). Quantum yield reflects the potential quantum efficiency of PSII, which is among the most susceptible components of photochemical processes and generally experiences the earliest impacts of stress conditions.^42^ During severe drought, Fv/Fm tends to decrease,^43^ indicating reduced photosynthesis or the occurrence of photoinhibition under stress.

Like other compounds, carotenoids typically increase during drought conditions to combat oxidative stress.^44,45^ This increase helps preserve the photosynthetic machinery longer, as observed in the MS-SEA treatment. Carotenoids act as antioxidants, neutralizing reactive oxygen species (ROS) that can damage lipids, proteins, and DNA. Additionally, carotenoids aid in dissipating excess energy as heat, thereby protecting photosystem II (PSII) from photodamage.^46^

In this context, carotenoids are among the most important indicators used to assess drought resistance in higher plants.^47^ For example, Parida et al.^48^ demonstrated that the degree of carotenoid reduction in cotton plants under drought conditions was greater in the sensitive genotype than in the moderately tolerant one. Carotenoids are essential for assembling the light-harvesting complex and for nonradiative dissipation of excess energy, involving the conversion of violaxanthin to zeaxanthin.^49,50^ During drought, plants typically increase their endogenous levels of α-tocopherol and carotenoids to cope with oxidative stress. This suggests that the extract of *K. alvarezii* may mitigate the effects of water stress, reducing the need for a significant increase in the level of synthesized carotenoids. The higher accumulation of carotenoids in water-deficient plants is an adaptation to enhance resilience to oxidative stress and protect photosynthetic systems. A recent study investigated the effect of a biostimulant on fruits and leaves, and through a metabolomic approach, it was shown that carotenoids were among the most affected metabolites. The application of the biostimulant increased the carotenoid content, suggesting activation of the enzymes responsible for their biosynthesis.^51^ In the case of treatments with biostimulants such as algal extracts, stress mitigation may alter this dynamic, indicating that the antioxidant mechanisms activated by the biostimulant could reduce the need for high levels of carotenoids, potentially delaying the activation of this pathway.

The increase in total soluble sugar concentration in the treatment with the aqueous algal extract (SS-SEA) suggests an effective response to water stress. This indicates that the plant can maintain or even enhance the biosynthesis of those essential primary metabolites that are essential for survival and adaptation to stress. Sugars are linked to drought tolerance, as they can accumulate, giving rise to intracellular osmotic resistance and preventing membrane rupture.^52^ Conversely, the SS-C treatment exhibited the lowest amount of total soluble sugars (TSS), i.e., 47.51 mg/g, suggesting that tomato metabolism under 50% water limitation may be less effective in enhancing the production of glucose, fructose, and disaccharides (e.g., sucrose) under stress conditions. However, the high contents of total starch (TS) observed in the SS-C treatment may indicate a strategy of driving glucose toward the biosynthesis of polysaccharides, in this case, starch. This lower accumulation of soluble sugars could limit the rapid stress response. In contrast, treatments with algae extract appear to promote a more efficient biochemical strategy under the same conditions, favoring a higher accumulation of soluble sugars as an adaptive response to stress.

This contrast between soluble sugar accumulation and starch storage reflects distinct drought-response strategies: while soluble sugars support rapid osmotic adjustment and stress signaling, starch accumulation represents a less flexible carbon allocation strategy under acute stress.

Soluble sugars and sucrose act as osmoregulators and energy sources, while glucose and fructose modulate energy metabolism and maintain redox homeostasis.^53^ Raffinose and stachyose provide protection for membranes and proteins against oxidative damage. Thus, the relationship between the experimental results and carbohydrate metabolism parameters suggests that the *K. alvarezii* aqueous extract enhanced the plant’s ability to respond to water stress. This could positively influence the aerial parts by promoting greater accumulation of soluble sugars, which serve as readily available energy reserves.^54^

On the other hand, algae-treated plants (FC-SEA, MS-SEA, and SS-SEA) exhibited lower total soluble sugars (TSS) compared to the SS-C treatment, indicating a metabolic strategy in response to stress that favors sugar accumulation over starch storage, in contrast to their respective controls under the same conditions. Shukla et al.^55^ investigated the use of algal extracts as biostimulants and demonstrated that their application can enhance drought resistance in plants. The study indicated that algal extracts contain various bioactive compounds that play crucial roles in modulating the antioxidant response and regulating osmolytes such as the soluble sugars sucrose, glucose, and fructose. These compounds contribute to increased antioxidant activity and improved osmotic balance by elevating osmolyte levels, facilitating cellular pressure maintenance, and enhancing membrane stability. Additionally, they modulate hormonal signaling by influencing the action of key drought-responsive hormones such as auxins, cytokinins, and abscisic acid. Thus, algae extracts promote metabolic and physiological adaptations that enable plants to maintain higher photosynthetic efficiency and overall performance, even under stress conditions.^56^

The results found regarding the phenolic compounds appear to be primarily related to the conditions of water availability to which the plants were subjected. Specifically, the quantity of these compounds was greater in plants not exposed to water limitation. Water stress can inhibit the expression of genes involved in the shikimic acid and phenylpropanoid pathways, thereby reducing the biosynthesis of phenolic compounds.^57^ Furthermore, the application of biostimulants may redirect plant metabolism, prioritizing carbon allocation for growth and maintenance processes instead of secondary metabolite production.^58^ Another possibility is that plants with lower amounts of these compounds may have utilized them to neutralize reactive oxygen species (ROS), a physiological condition that increases under abiotic stress.

Thus, the observed reduction in phenolic and flavonoid contents under drought does not necessarily indicate reduced metabolic activity but rather an increased turnover of these compounds in response to enhanced oxidative pressure.

Phenols and flavonoids act as natural antioxidants, protecting cells from oxidative damage. In plant metabolism, polyphenols play a crucial role in responding to oxidative stress caused by adverse conditions, such as drought, salinity, and heavy metal exposure. After neutralizing ROS, polyphenols can follow different metabolic pathways, such as conversion into quinones, which can bind to proteins and participate in redox reactions. Additionally, they may be conjugated with sugars or amino acids, making them less reactive and allowing for their storage in vacuoles to prevent cellular damage. Studies have shown that the application of biostimulants can induce a transient increase in antioxidant demand, leading to an accelerated consumption of phenolic compounds and flavonoids.^59,60^ In the study by Cerrutti et al.,^61^ the observed modulation suggests that the biostimulant not only adjusts enzymatic and nonenzymatic defenses but also stimulates the production of phenolic compounds, which are secondary metabolites with well-known antioxidant properties. Moreover, transcriptomic analysis revealed that the *Ascophyllum nodosum*-based biostimulant increases the expression of genes involved in transcriptional regulation, kinase signaling, redox homeostasis, and defense mechanisms, including the phenylpropanoid pathway, which is associated with the biosynthesis of secondary metabolites like phenolics.

Given the interconnected nature of morphological, physiological, and biochemical traits, multivariate analysis provides an integrated perspective of how drought stress and biostimulant application reshape plant performance as a whole.

Analyzing the PCA results for morphophysiological and biochemical parameters, it is evident that the best responses were observed with the application of SEA under moderate (MS) and severe (SS) stress conditions. This indicates enhanced energetic metabolic activity, as demonstrated by the accumulation of TCN in the MS-SEA treatment and TSS and TC in the SS-SEA treatment, reflecting a stress response. Consequently, it appears that the metabolic pathways may vary depending on the intensity of water stress, leading to the accumulation of distinct classes of metabolites.

The combined analysis of the morphophysiological and biochemical PCAs demonstrates that the most pronounced responses occurred in treatments receiving the aqueous extract of *K. alvarezii* (SEA) under both moderate (MS) and severe (SS) water stress conditions. These responses indicate enhanced energetic metabolic activity, evidenced by the accumulation of TCN in the MS-SEA treatment and of TSS and TC in the SS-SEA treatment, suggesting that different levels of water stress activate distinct metabolic pathways, leading to the accumulation of specific classes of metabolites.

Furthermore, the treatment-based PCA evaluation reveals that MS-SEA clustered with key morphological parameters, such as plant height, number of fruits, and number of flowers, as well as with carotenoids, indicating a multivariate association between vegetative growth and secondary metabolism. In contrast, SS-SEA clustered with total carbohydrates and total soluble sugars, which represent primary metabolites directly involved in stress responses. Together, these findings indicate that the algae extract promotes a metabolic reorganization adjusted to the intensity of water stress, contributing to the maintenance of plant performance under both moderate and severe excess-water conditions.

These findings align with previous evidence highlighting the importance of carbohydrate metabolism in plant tolerance to water deficits. Thomas et al.^62^ showed that drought-tolerant rice genotypes accumulate more sucrose in leaves and phloem by upregulating sucrose synthase (SuSy) and sucrose transporters (SUC2), thereby improving osmoprotection and membrane integrity. They also demonstrated that tolerant plants redirect sucrose to the roots and activate invertase and amylases to sustain energy metabolism under stress. The increased soluble sugars and total carbohydrates observed in SEA-treated plants, especially under severe stress, follow this same physiological pattern, indicating that the *K. alvarezii* extract may enhance carbohydrate-based protective responses. This supports the hypothesis that improved sugar metabolism contributes to the superior performance of treated plants under excess-water stress.

Trivedi et al.^63^ highlight that the *K. alvarezii* biostimulant promotes positive effects in plants, ranging from seed germination and vigor to plant growth and yield. Furthermore, the authors reference studies that emphasize resilience to stress in various species, such as maize and wheat. The *K. alvarezii* biostimulant can regulate genes associated with stress responses, defense mechanisms, phytohormones, nitrogen metabolism, signal transduction, photosynthesis, ion transport, antioxidant pathways, and polysaccharide metabolism. However, despite these promising findings, there remain significant gaps in our understanding of the underlying mechanisms and long-term effects resulting from the *K. alvarezii* aqueous extract application. As such, further research is essential to fully elucidate its potential and optimize its use in agricultural practices. Metabolically, both the hypothesis of increased consumption of phenolic compounds and flavonoids under stress and the hypothesis of reduced synthesis are plausible and not mutually exclusive. The extent of these changes depends on the level of stress, the antioxidant capacity of plants, and the mechanisms of action of biostimulants. Complementary analyses, such as the activity of antioxidant enzymes (SOD, CAT, APX) and the expression of genes involved in the biosynthesis of phenolic compounds, could provide deeper insights into the mechanisms underlying the results herein described.

Overall, the results demonstrate that drought stress triggers a coordinated network of metabolic and physiological responses in tomato plants and that the application of *K. alvarezii* extract modulates these responses by enhancing metabolic flexibility, photoprotection, and carbohydrate-based stress mitigation. Rather than acting on a single trait, the biostimulant promotes an integrated adjustment of plant metabolism that supports acclimation across increasing levels of water deficit.

## 5 Conclusions

This study demonstrates that the application of the *Kappaphycus alvarezii* extract enhances tomato plant adaptation to water deficit by promoting coordinated metabolic and physiological adjustments rather than isolated trait modifications. The most notable responses involved the modulation of carbohydrate metabolism and photoprotective mechanisms, particularly the accumulation of soluble sugars and carotenoids, which play central roles in osmotic adjustment, energy balance, and protection of the photosynthetic apparatus under drought conditions.

Multivariate analysis further revealed that the biostimulant induced distinct metabolic reprogramming depending on stress intensity, supporting the concept that stress-adaptive responses are dynamically regulated rather than uniform across water deficit levels. These findings advance the current understanding of algal-based biostimulants by highlighting their role in enhancing metabolic flexibility, a key component of plant resilience to abiotic stress.

In line with current perspectives in the biostimulant field, these results reinforce the view that biostimulants should be regarded as functional tools within plant physiological management rather than as inputs aimed solely at short-term yield gains. Their value lies in stabilizing plant function, reducing sensitivity to stress, and supporting adaptive responses under variable environmental conditions. In this context, the effectiveness of the *K. alvarezii* extract observed here underscores the importance of aligning mechanistic understanding with agronomic application, regulatory frameworks, and realistic performance expectations.

Future research should therefore move beyond isolated response indicators and focus on integrated system-level approaches to biostimulant evaluation. Elucidating the molecular and biochemical mechanisms underlying these responses, particularly those involving carbohydrate metabolism, antioxidant systems, and stress-related signaling pathways, through targeted metabolomics, transcriptomics, and enzyme activity assays will be essential. Long-term and field-scale studies will further support the responsible integration of *K. alvarezii*-based biostimulants into sustainable and adaptive agricultural strategies.
